# Waist-to-height ratio, body fat, and macronutrient intake as predictors of lipid abnormalities in elite Turkish athletes: a comparative study

**DOI:** 10.7717/peerj.20743

**Published:** 2026-02-10

**Authors:** Funda Tamer, Betul Kisioglu Halis, Pelin Bilgic

**Affiliations:** 1 Department of Nutrition and Dietetics/Faculty of Health Sciences, Hacettepe University, Ankara, Turkey; 2 Department of Nutrition and Dietetics/Faculty of Health Sciences, Duzce University, Duzce, Turkey

**Keywords:** Athletes, Diet, Endurance exercise, Lipid profile, Strength exercise

## Abstract

**Background:**

Regular physical activity can improve the blood lipid profile, yet athletes may still experience dyslipidemia. This study examined lipid profiles in Turkish endurance and strength athletes in relation to the dietary intake.

**Methods:**

Eighty-four participants, including strength athletes (*n* = 45), endurance athletes (*n* = 20), and non-athletes (*n* = 19) were assessed for dietary intake (quantitative food-frequency questionnaire), body composition, and blood lipid profile.

**Results:**

Endurance athletes had a lower body mass index (BMI), body fat (%), fat mass, waist-to-hip ratio, and waist-to-height ratio than strength athletes and non-athletes (*p* < 0.05). Endurance athletes derived a lower percentage of daily energy intake from protein and fat, a higher from carbohydrate, and consumed more dietary fiber (*p* < 0.05). Compared with endurance athletes, strength athletes showed higher serum low-density lipoprotein cholesterol (LDL-C) and apolipoprotein B (ApoB) levels, total cholesterol/high-density lipoprotein cholesterol (TC/HDL-C), LDL-C/HDL-C, and ApoB/ApoA-1 ratios, a higher atherogenic index, and lower levels of HDL-C and ApoA-1 (*p* < 0.05). Overall, athletes had lower serum triacylglycerol (TG), very low-density lipoprotein cholesterol (VLDL-C), and higher LDL-C levels than non-athletes (*p* < 0.05). Logistic regression models showed that waist-to-height ratio and body fat (%) were consistent predictors of adverse lipid outcomes, independent of dietary energy intake; strength athletes had higher odds of elevated LDL-C and ApoB, highlighting distinct lipid risks by sport group.

**Conclusion:**

Endurance athletes displayed a more favorable lipid profile than strength athletes and non-athletes. Group differences in lipids likely reflect a combination of adiposity, dietary patterns, and sport-specific behaviors.

## Introduction

Epidemiological studies in adults show that regular physical activity and high aerobic fitness improve cardiovascular risk factors, including blood lipids (Guo et al., 2011; Mazloom, Salehi & Eftekhari, 2008). Higher volume and/or intensity of aerobic physical activity, performed regularly over time, is associated with lower atherosclerosis risk, higher high-density lipoprotein cholesterol (HDL-C) (Kodama et al., 2007), and lower total cholesterol (TC), low-density lipoprotein cholesterol (LDL-C) (Lira et al., 2010), and triacylglycerol (TG) levels (Boraita, 2004). However, the combined influence of sport type, long-term training effects, and diet on lipid metabolism in athletes has received limited attention.

Despite high physical activity levels, athletes can still experience dyslipidemia, a risk factor for atherosclerosis, yet data on this topic remain scarce (Di Gioia et al., 2024). Evidence from endurance athletes (cross-country skiers, road cyclists) (Lippi et al., 2006) and from multi-sport Olympic cohorts spanning endurance and non-endurance (power/strength, skills, mixed) disciplines (Di Gioia et al., 2024; Pelliccia et al., 2017) shows that athletes have a noteworthy prevalence of cardiovascular risk factors, including dyslipidemia. Identification and management of dyslipidemia in athletes has become a growing clinical concern and should not be underestimated (Di Gioia et al., 2024).

Blood lipids correlate with dietary intake, and body composition (Mansour et al., 2024). Higher body fat percentage is associated with higher TG and LDL-C levels and lower HDL-C levels (Petridou, Lazaridou & Mougios, 2005). Low dietary fat intake may help explain the normal plasma lipid and lipoprotein profiles observed in physically active women (Mazloom, Salehi & Eftekhari, 2008). In contrast, the hypercholesterolemic profiles observed in some ultra-endurance athletes may be linked to high consumption of saturated fat and cholesterol, combined with low fiber intake (Grundy et al., 2004).

In summary, there are limited studies investigating the relationship between dietary intake, body composition and blood lipid levels in athletes. Current evidence highlights the need to monitor athletes for abnormal lipid profiles and future atherosclerotic risk. Existing studies often lack comparisons across athlete groups and non-athletes and have limited demographic diversity. Therefore, we examined dietary intake, body composition, and lipid profiles in athletes from different sport disciplines *vs*. non-athletes in Türkiye.

## Materials and Methods

### Participants

This study included Turkish national male strength athletes (29 wrestlers and 16 weightlifters (*n* = 45)) and endurance athletes (15 runners and five cyclists (*n* = 20)). Athletes were recruited through coordination with national teams and the Turkish Ministry of Youth and Sports. Seventeen participants were excluded due to training schedules incompatibility, refusal to undergo anthropometric measurements, or noncompliance with pre-blood draw instructions. The control group comprised 19 individuals (out of 84 total participants) who were age-matched non-athletes and had a similar body mass index (BMI) compared to the athlete groups, with an average age of 24 years and a BMI of 24.65 kg/m^2^ (range: 21.5–33.0). Those engaging in any regular or occasional physical activity during the past year were excluded. All participants were healthy and free from metabolic disorders. Individuals using prescription medications, herbal remedies, or supplements affecting lipid profiles were excluded.

The study was approved by the Ethics Committee of Hacettepe University, Ankara, Türkiye, (Number: 01 02 402 001) in compliance with the principles outlined in the Declaration of Helsinki. Informed, written consent was obtained from all subjects.

### Study design

**Figure 1:**
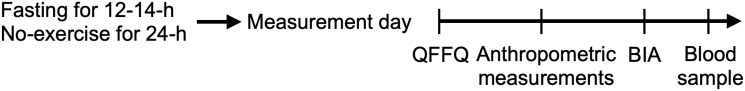
Study design. All subjects attended the laboratory in condition with 12–14-h of fasting and 24-h with no exercise at the measurement day. A standard questionnaire was performed including a QFFQ for dietary assessment. Anthropometric measurements and BIA were performed. Venous blood samples were drawn from all subjects for the determination of fasting glucose and lipid profile. BIA, multi-frequency bioelectrical impedance analyzer; QFFQ, quantitative food frequency questionnaire.

The study design is shown in Fig. 1. After confirming eligibility, data were collected during a single laboratory visit. Participants arrived after 12–14-h of fasting and at least 24-h without exercise. General information (age, past and/or current smoking status, daily physical activity levels, duration of regular training, and education status) was collected *via* a standardized questionnaire administered by a trained dietitian. The visit also included a dietary assessment, anthropometric measurements, bioelectrical impedance analysis (BIA), and blood sampling (Fig. 1).

### Dietary assessment

As previously described (Devrim-Lanpir et al., 2020), a validated quantitative food frequency questionnaire (QFFQ) assessed usual intake over the prior 3 months (foods, amounts, and frequencies). The QFFQ included 110 food items. A dietitian administered the QFFQ in face-to-face interviews. A photographic Meal and Food Atlas was used to record the quantity and portion size of food items more accurately (Gunes et al., 2015). From the dietary assessment we derived energy (kcal/day and kcal/kg), protein (% of energy, g/kg), fat (% of energy, g/kg), carbohydrate (% of energy, g/kg) intakes and carbohydrate protein ratio (CHO:PRO).

### Anthropometric measurements

Height was measured to 0.1 cm on a wall-mounted stadiometer: weight to 0.1 kg, fasting and without shoes. BMI was calculated as weight (kg)/height (m^2^). Waist-to-hip ratio (WHR) was calculated as waist circumference divided by hip circumference; each was measured twice to 0.1 cm and averaged. Waist-to-height ratio (WHtR) was calculated as waist circumference divided by height.

### Body composition

The body composition of the participants was examined by two methods: (1) multi-frequency BIA (TANITA MC-780; Tokyo, Japan; 0.1 kg accuracy) (Devrim-Lanpir et al., 2019) to estimate body fat percentage (%, BIA), fat mass (kg), fat-free mass (kg), and total body water (L); and (2) skinfold thickness measurements to estimate body fat percentage (%, skinfold) using the Brozek equation (Devrim-Lanpir et al., 2021).

### Sample collection and preparation

Venous blood was collected by antecubital venipuncture of the right arm after the fasting and exercise-free period. The blood samples were immediately centrifuged for 12 min at 4,000 g, and serum was collected. Blood samples were analyzed immediately (for serum glucose and serum lipid profile markers).

### Determination of fasting glucose and lipoprotein profile in blood

Serum glucose (Glucose GOD-PAP; Roche/Hitachi Diagnostics), TC (CHOD-PAP; Roche/Hitachi Diagnostics), TG (triglyceride/GB GPO-PAP; Roche/Hitachi Diagnostics) and HDL-C (HDL-C plus; Roche/Hitachi Diagnostics, 904/911/912/917/MOD: ACN 035) were measured with enzymatic color assays according to manufacturers’ instructions using an absorbance microplate reader (EL808; Biotek, Winooski VT, USA) in the Biochemistry Laboratory of Hacettepe University Hospital (Kirkpantur et al., 2008). After HDL-C determination, LDL-C and very low-density lipoprotein cholesterol (VLDL-C) were estimated by the Friedewald equation. Briefly, LDL-C was calculated as (TC)–(HDL-C)–(TG/5); VLDL-C was calculated as (TG/5). Their concentrations were expressed in mg/dL (Friedewald, Levy & Fredrickson, 1972).

Serum ApoA-1, ApoB and lipoprotein (a) [Lp(a)] were measured by nephelometry (Beckman IMMAGE Immunochemistry Systems) according to manufacturers’ instructions in the Biochemistry Laboratory of Hacettepe University Hospital (Yildirir et al., 2001).

Impaired fasting glucose was defined as ≥100 mg/dL (5.6 mmol/l) (American Diabetes Association, 2013). Lipid categories followed (Grundy et al., 2004): TC-desirable, <200 mg/dL; borderline high, between 200 and 239 mg/dL; and high, ≥240 mg/dL. TG-normal, <150 mg/dL; borderline high, between 150 and 199 mg/dL; and high, ≥200 mg/dL. LDL-C-optimal, <100 mg/dL; near optimal, between 100 and 129 mg/dL; borderline high, between 130 and 159 mg/dL; and high, ≥160 mg/dL. HDL-C-high, ≥60 mg/dL; and low, <40 mg/dL for men (Grundy et al., 2004).

We calculated the frequencies of athletes and non-athletes at risk for dyslipidemia (per cut-offs) for each lipid category. In addition to clinical cut-off points, we used: ApoA-1 < 115 mg/dL (Walldius & Jungner, 2004), ApoB > 100 mg/dL (Walldius & Jungner, 2004), Lp(a) > 30 mg/dL (Kostner et al., 1981), TC/HDL-C > 5.0, LDL-C/HDL-C > 3.0 (Kubalová et al., 2025; Salcedo-Cifuentes et al., 2020), and ApoB/ApoA-1 ≥ 0.90 (Walldius & Jungner, 2006). We also calculated the abnormal marker count (*n ≥ 1*) if any of TC, TG, LDL-C, or HDL-C exceeded or fell below their reference cut-offs (Grundy et al., 2004).

The Atherogenic Index of Plasma was calculated as the base-10 logarithm of the ratio of plasma TG concentration to HDL-C concentration, both expressed in molar units: log_10_(TG/HDL-C). The atherogenic index is a validated marker of plasma atherogenicity and cardiovascular risk, with cut-off points defined as: low risk (<0.11), moderate risk (0.11–0.21), and high risk (>0.21) (Dobiášová et al., 2011).

### Statistical analysis

The elite athletes in this study included European, World Championship, and multiple national title holders. Over 70% of the athletes in the training camp participated; thus, the dataset closely represents that population rather than a small convenience sample. Non-athlete participants were selected by stratified sampling to match athletes on age and BMI.

Before the analysis, normality was assessed with the Shapiro–Wilk test. Results are expressed as mean ± standard deviation (SD). Depending on distribution, we used parametric one-way analysis of variance (ANOVA) or non-parametric Kruskal Wallis tests to compare the three groups. *Post-hoc* tests were Tukey (ANOVA) or Mann–Whitney U (pairwise non-parametric). Within athlete groups, relationships were examined with Pearson and Spearman correlations. Correlations effect sizes were classified by |r|: trivial <0.10, small 0.10–0.29, moderate 0.30–0.49, large ≥0.50 (Cohen, 2013); direction is indicated by the sign of r. To limit false discoveries within each correlation matrix, we controlled the false discovery rate (Benjamini–Hochberg) at q < 0.05 within each table. Tables display raw *p*-values; cells remaining significant after false discovery rate are shown in bold.

Based on correlations, energy intake (kcal/day), CHO:PRO, fiber (g/1,000 kcal), WHtR, and study group were included in binary logistic regressions for eight lipid outcomes, using a median split to define high *vs*. low levels. Each lipid was split at the sample median to balance case numbers and avoid sparse-cell instability where lipid thresholds are lacking. For regression, WHtR values were multiplied by 100 to improve odds ratio (OR) interpretability without changing effect sizes. Logistic regression analysis estimated the ORs and 95% confidence intervals (CIs). Multicollinearity was assessed *via* variance inflation factors (VIF < 5) and tolerance (>0.20) from linear regression with the same covariates. To avoid collinearity, energy and macronutrient intakes (g/kg) were not entered in the same model. For logistic regressions, Benjamini–Hochberg false discovery rate (BH FDR) (q < 0.05) was applied within each model to control multiple testing. Analyses were performed in SPSS (version 23.0; SPSS Inc., Chicago, IL, USA). Statistical significance was set at *p* < 0.05.

## Results

### Characteristics and body composition

A total of 84 males participated: 45 elite strength athletes (29 wrestlers, 16 weightlifters), 20 endurance athletes (15 runners, five cyclists), and 19 non-athletes. Mean age was 23.3 ± 3.5 years for athletes and 25.9 ± 5.9 years for the non-athletes. Groups were frequency-matched on fat-free mass and age. There were no between group differences in marital status, educational status, age, smoking status (*p* > 0.05; Supplemental Data File). These similar demographics support the sustainability of the control group.

Body composition and anthropometric measurements for all groups are shown in Table 1. Endurance athletes had a lower BMI (20.2 ± 0.9 kg/m^2^), body fat percantage (5.8 ± 3.1%, BIA, skinfold), and fat mass (3.4 ± 1.8 kg) than strength athletes (28.0 ± 3.5 kg/m^2^, 13.2 ± 4.5% and 11.1 ± 6.3 kg, respectively) and non-athletes (26.2 ± 5.1 kg/m^2^, 19.0 ± 7.2% and 16.4 ± 9.0 kg, respectively), alongside differences in fat-free mass and total body water (*p* < 0.05). Although BMI did not differ between strength athletes and non-athletes (*p* > 0.05), strength athletes had lower body fat (%, BIA, skinfold), and fat mass (*p* < 0.05). Waist circumference was 72.8 ± 2.3 cm in endurance athletes, 84.8 ± 9.6 cm in strength athletes, and 88.8 ± 9.9 cm in non-athletes (*p* < 0.05). Furthermore, WHR was 0.81 ± 0.04 in endurance athletes, 0.85 ± 0.04 in strength athletes, and 0.90 ± 0.06 in non-athletes (*p* < 0.05). Waist-to-height ratio (WHtR) was 0.43 ± 0.01 in endurance athletes, 0.50 ± 0.04 in strength athletes, and 0.51 ± 0.06 in non-athletes (*p* < 0.05) (Table 1).

**Table 1:** Body compositions and anthropometric measurements of strength athletes, endurance athletes, and non-athletes.

Measurements^†^	Strength athletes			Endurance athletes				
	Wrestlers (*n* = 29)	Weightlifters (*n* =16)	Total (*n* = 45)	Runners (*n* = 15)	Cyclists (*n* = 5)	Total (*n* = 20)	Non-athletes (*n* = 19)	*p*
Weight (kg)	81.4 ± 19.0	78.5 ± 13.8	80.3 ± 17.2^a^	58.9 ± 3.3	59.3 ± 5.1	59.6 ± 3.9^b^	79.5 ± 14.2^a^	<0.001
Height (cm)	169.2 ± 9.3	166.9 ± 8.8	168.4 ± 9.1^a^	170.6 ± 2.6	173.8 ± 4.1	171.4 ± 3.2^a^	174.6 ± 5.6^b^	0.013
BMI (kg/m^2^)	28.0 ± 3.8	28.1 ± 3.0	28.0 ± 3.5^a^	20.2 ± 0.9	20.1 ± 1.1	20.2 ± 0.9^b^	26.2 ± 5.1^a^	**<0.001**
Body fat (%, skinfold)	13.2 ± 5.2	13.0 ± 3.0	13.2 ± 4.5^a^	5.3 ± 3.2	7.6 ± 3.0	5.8 ± 3.1^b^	19.0 ± 7.2^c^	**<0.001**
Body fat (%, BIA)	13.0 ± 5.2	13.7 ± 3.1	13.2 ± 4.5^a^	5.3 ± 3.2	7.5 ± 2.5	5.8 ± 3.1^b^	19.0 ± 7.2^c^	0.074
Fat mass (kg)	11.5 ± 7.2	10.4 ± 4.3	11.1 ± 6.3^a^	3.1 ± 1.8	4.6 ± 1.9	3.4 ± 1.8^b^	16.4 ± 9.0^c^	**<0.001**
Fat-free mass (kg)	69.9 ± 12.4	68.0 ± 10.1	69.2 ± 11.5^a^	55.7 ± 3.9	54.8 ± 4.1	55.6 ± 3.8^b^	63.1 ± 6.3^a^	**<0.001**
Total body water (L)	51.1 ± 9.0	49.8 ± 7.4	50.6 ± 8.4^a^	40.8 ± 2.8	40.1 ± 3.0	40.7 ± 2.8^b^	46.2 ± 4.6^a^	**<0.001**
Waist circumference (cm)	85.8 ± 10.6	83.3 ± 7.7	84.8 ± 9.6^a^	72.6 ± 2.1	73.5 ± 3.1	72.8 ± 2.3^b^	88.8 ± 9.9^a^	**<0.001**
WHR	0.86 ± 0.03	0.83 ± 0.03	0.85 ± 0.04^a^	0.81 ± 0.03	0.82 ± 0.06	0.81 ± 0.04^b^	0.90 ± 0.06^c^	**<0.001**
WHtR	0.51 ± 0.04	0.50 ±0.03	0.50 ± 0.04^a^	0.43 ±0.01	0.43 ±0.03	0.43 ± 0.01^b^	0.51 ± 0.06^a^	**<0.001**

**Notes:**

BIA, bioelectrical impedance analysis; BMI, body mass index; WHR, waist-to-hip ratio; WHtR, waist-to-height ratio.

Comparisons between strength athletes, endurance athletes and non-athletes (*p* < 0.05, Kruskal–Wallis test/one-way ANOVA test).

Pairwise Mann–Whitney tests were adjusted for multiple comparisons within each variable using the Holm–Bonferroni method.

† Data is expressed as mean ± SD.

^a, b, c^No significant difference between groups containing the same letter (*p* < 0.05, Mann–Whitney U test/Tukey *post-hoc* test).

Cells remaining significant after false discovery rate are shown in bold.

### Dietary intake

Energy and macronutrient intakes are presented in Table 2. Median energy intake was higher in endurance athletes (110.1 (63.9–159.9) kcal/kg) than in strength athletes (42.3 (32.9–52.9) kcal/kg) and non-athletes (37.4 (33.6–41.3) kcal/kg) (*p* < 0.001). According to the American Dietetic Association, 45–65% of total energy should come from carbohydrate, 10–35% from protein, and 20–35% of energy from fat (Rodriguez, DiMarco & Langley, 2009). Thus, weightlifters (35.2 (29.3–38.2) %) and non-athletes (36.2 (29.3–41.1) %) had slightly higher dietary fat as a percentage of energy than recommended. Percent energy from fat was significantly lower in endurance athletes (30.3 (26.8–34.4) %) than in strength athletes (35.2 (29.3–38.2) %) (*p* < 0.05). However, endurance athletes had a higher median fat intake (3.8 (2.0–5.1) g/kg) than strength athletes (1.7 (1.4–2.0) g/kg) and non-athletes (1.6 (1.1–2.0) g/kg) (*p* = 0.001). Although percent energy from protein did not differ significantly among groups, endurance athletes had a higher median protein intake (3.1 (1.8–4.0) g/kg) than strength athletes (1.3 (1.1–1.2) g/kg) and non-athletes (1.4 (1.0–1.6) g/kg) (*p* = 0.001). Endurance athletes had higher carbohydrate intake (58.5 (54.2–62.9) %, 15.5 (9.3–25.5) g/kg) than strength athletes (51.9 (46.6–56.9) %, 5.9 (3.7–7.6) g/kg) (*p* < 0.05) and did not differ from non-athletes (49.9 (42.9–60.9) %, 4.5 (4.0–5.9) g/kg). The median CHO:PRO ratio was higher in endurance athletes (5.2 (4.7–5.9)) than in strength athletes (4.1 (3.4–4.9)), and non-athletes (4.5 (2.8–5.3)) (*p* = 0.003). There were no significant differences in any median dietary intakes between strength athletes and non-athletes (Table 2).

**Table 2:** Energy, macronutrient, and fiber intakes of strength athletes, endurance athletes, and non-athletes.

Dietary intakes^†^	Strength athletes			Endurance athletes				
	Wrestlers (*n* = 29)	Weightlifters (*n* = 16)	Total (*n* = 45)	Runners (*n* = 15)	Cyclists (*n* = 5)	Total (*n* = 20)	Non-athletes (*n* = 19)	*p*
Energy (kcal/day)	3,012.2 (2,455.5–4,128.9)	4,605 (3,150–9,018)	3,514 (2,739–4,563) ^a^	7,012 (4,005–11,473)	3,854 (3,273–4,926.6)	5,861.5 (3,485.5–9,520.8) ^b^	2,636 (2,399–4,051)^ab^	**0.006**
Energy (kcal/kg)	39.1 (29.3–45.9)	65.5 (45.7–114.6)	42.3 (32.9–52.9) ^a^	116.7 (72.0–184.5)	32.1 (22.1–48.9)	110.1 (63.9–159.9)^b^	37.4 (33.6–41.3)^a^	**<0.001**
Protein (% of energy)	12.0 (10.9–14.5)	13.2 (11.2–14.2)	12.6 (11.2–14.3)	11.0 (10.4–11.8)	11.3 (10.7–11.9)	11.0 (10.6–11.9)	13.2 (10.9–15.8)	**0.042**
Protein (g/kg)	1.1 (1.0–1.4)	2.2 (1.3–3.9)	1.3 (1.1–1.2) ^a^	3.4 (2.1–4.1)	1.2 (0.6–1.7)	3.1 (1.8–4.0)^b^	1.4 (1.0–1.6)^a^	**<0.001**
Fat (% of energy)	34.7 (31.1–41.8)	35.2 (29.3–38.2)	35.0 (29.6–40.2) ^a^	30.8 (27.4–34.2)	24.0 (20.5–27.3)	30.3 (26.8–34.4)^b^	36.2 (29.3–41.1)^ab^	**0.040**
Fat (g/kg)	1.4 (1.3–1.7)	2.8 (1.6–4.1)	1.7 (1.4–2.0) ^a^	4.2 (3.0–5.8)	0.9 (0.7-1.5)	3.8 (2.0–5.1)^b^	1.6 (1.1–2.0)^a^	**0.001**
Carbohydrate (% of energy)	51.9 (45.9–56.6)	52.5 (47.8–57.7)	51.9 (46.6–56.9) ^a^	58.5 (54.3–62.7)	60.0 (54.0–66.5)	58.5 (54.2–62.9)^b^	49.9 (42.9–60.9)^ab^	**0.005**
Carbohydrate (g/kg)	5.2 (3.6–6.3)	8.3 (5.9–13.6)	5.9 (3.7–7.6) ^a^	16.0 (10.6–30.5)	4.8 (3.0–7.2)	15.5 (9.3–25.5)^b^	4.5 (4.0–5.9)^a^	**<0.001**
CHO:PRO	4.2 (3.4–5.2)	4.0 (3.6–4.8)	4.1 (3.4–4.9) ^a^	5.3 (4.7–5.9)	5.1 (4.0–5.5)	5.2 (4.7–5.9)^b^	4.5 (2.8–5.3)^a^	**0.003**
Fiber (g/day)	4.9 (3.3–6.6)	8.6 (3.8–14.0)	5.5 (3.6–10.9) ^a^	11.5 (7.4–15.7)	2.8 (0.6–6.7)	10.6 (6.2–15.0)^b^	4.9 (2.6–7.4)^a^	**0.021**
Fiber (g/1,000 kcal)	1.8 (1.4–2.4)	1.9 (0.9–2.0)	1.7 (1.2–2.4)	1.6 (1.5–2.0)	1.4 (0.4–1.5)	1.5 (1.4–2.0)	1.5 (1.0–2.4)	0.636
*Participants meeting fiber* *requirements*								
*g/day*	–	2 (12.5)	2 (4.4)	1 (6.7)	1 (20.0)	2 (10.0)	1 (5.3)	0.827
*g/1,000 kcal*	–	–	–	–	–	–	1 (5.3)	0.228

**Notes:**

CHO:PRO, carbohydrate:protein ratio.

Comparisons between strength athletes, endurance athletes and non-athletes (*p* < 0.05, Kruskal–Wallis test).

Pairwise Mann–Whitney tests were adjusted for multiple comparisons within each variable using the Holm–Bonferroni method.

Participants meeting fiber requirements: Values are *n* (%) of participants above cut-offs (EFSA (≥25 g/day; ≥14 g/1,000 kcal)) (EFSA Panel on Dietetic Products, Nutrition, and Allergies, 2010). P: Fisher’s exact test (Monte-Carlo) for 2 × 3 tables when any expected cell <5; otherwise Pearson χ^2^.

† Data is expressed as median (IQR = 25^th^–75^th^).

^a, b, c^No significant difference between groups containing the same letter (*p* < 0.05, Mann–Whitney U test).

Cells remaining significant after false discovery rate are shown in bold.

Additionally, endurance athletes had a higher median fiber intake (10.6 (6.2–15.0) g/day) than strength athletes (5.5 (3.6–10.9) g/day) and non-athletes (4.9 (2.6–7.4) g/day) (*p* = 0.021). The European Food Safety Authority (EFSA) cut-offs (EFSA Panel on Dietetic Products, Nutrition, and Allergies, 2010), adopted in Türkiye (T.C. Sağlık Bakanlığı & Halk Sağlığı Genel Müdürlüğü, 2022), were applied as ≥25 g/day; ≥14 g/1,000 kcal to determine the number of participants meeting the fiber requirements. Participants meeting daily fiber requirement were 10% (*n* = 2) in endurance athletes, 4.4% (*n* = 2) in strength athletes, and 5.3% (*n* = 1) in non-athletes; proportions did not differ across groups (*p* > 0.05) (Table 2).

### Serum lipid profile

Fasting serum glucose and lipid profiles for the strength, endurance and non-athlete groups are presented in Table 3. Mean fasting serum glucose ranged from 67.9 to 74.9 mg/dL and was within reference values in all subgroups. No participant met criteria impaired fasting glucose (American Diabetes Association, 2013) (Table 3).

**Table 3:** Serum glucose and lipid profile of strength athletes, endurance athletes, and non-athletes.

Parameters^†^	Reference values^‡^ (mg/dL)	Strength athletes			Endurance athletes			Non-athletes (*n* = 19)	*p*
		Wrestlers (*n* = 29)	Weightlifters (*n* = 16)	Total (*n* = 45)	Runners (*n* = 15)	Cyclists (*n* = 5)	Total (*n* = 20)		
Glucose	70–110	74.4 ± 6.3	67.9 ± 6.0	72.1 ± 6.9	73.3 ± 14.3	74.0 ± 12.0	73.4 ± 13.5	74.9 ± 6.2	0.213
TC	<200	182.5 ± 27.6	186.8 ± 30.4	184.0 ± 28.4	169.6 ± 20.8	144.8 ± 16.9	163.4 ± 22.3	178.5 ± 33.6	0.061
TG	<150	85.8 ± 37.4	106.4 ± 29.6	93.1 ± 36.0^a^	97.5 ± 55.3	57.0 ± 18.5	87.4 ± 51.4^a^	125.8 ± 58.7^b^	**0.006**
HDL-C	35–60	48.6 ± 13.6	21.0 ± 9.5	38.8 ± 18.1^a^	53.2 ± 10.4	56.4 ± 7.8	54.0 ± 9.7^b^	47.5 ± 8.7^a^	**0.002**
LDL-C	<130	117.8 ± 24.6	144.4 ± 29.2	127.2 ± 29.0^a^	96.9 ± 13.0	77.0 ± 15.5	92.0 ± 16.0^b^	105.9 ± 26.5^b^	**<0.001**
VLDL-C	<40	17.2 ± 7.5	21.3 ± 5.9	18.6 ± 7.2^a^	19.5 ± 11.1	11.4 ± 3.7	17.5 ± 10.3^a^	23.0 ± 11.6^b^	**0.036**
ApoA-1	115–210	119.9 ± 22.0	69.7 ± 26.1	102.0 ± 33.6^a^	144.8 ± 19.4	128.2 ± 9.2	140.6 ± 18.7^b^	121.8 ± 16.6^a^	**<0.001**
ApoB	55–135	84.8 ± 18.4	125.4 ± 19.8	99.2 ± 27.1^a^	75.2 ± 8.7	61.6 ± 10.1	71.8 ± 10.6^b^	86.4 ± 20.0^c^	**<0.001**
Lipoprotein (a)	0–30	13.7 ± 8.8	10.7 ± 2.9	12.6 ± 7.3	13.2 ± 5.6	19.6 ± 16.0	14.8 ± 9.3	26.5 ± 23.6	0.064
TC/HDL-C	*NA*	4.1 ± 1.7	10.6 ± 5.1	6.4 ± 4.6^a^	3.3 ± 0.6	2.6 ± 0.4	3.1 ± 0.6^b^	3.9 ± 0.9^ab^	**<0.001**
LDL-C/HDL-C	*NA*	2.6 ± 1.4	8.4 ± 4.4	4.7 ± 4.0^a^	1.9 ± 0.3	1.4 ± 0.3	1.7 ± 0.4^b^	2.3 ± 0.7^a^	**<0.001**
ApoB/ApoA-1	*NA*	0.75 ± 0.3	2.19 ± 1.3	1.26 ± 1.1^a^	0.52 ± 0.1	0.49 ± 0.1	0.52 ± 0.1^b^	0.71 ± 0.2^a^	**<0.001**
Atherogenic index	*NA*	0.23 ± 0.3	0.74 ± 0.2	0.41 ± 0.3^a^	−0.01 ± 0.2	0.22 ± 0.3	0.16 ± 0.2^b^	0.40 ± 0.2^a^	**0.003**

**Notes:**

TC, total cholesterol; TG, triglycerides; HDL-C, high-density lipoprotein cholesterol; LDL-C, low-density lipoprotein cholesterol; VLDL-C, very low-density lipoprotein cholesterol; ApoA-1, Apolipoprotein A-1; ApoB, Apolipoprotein B; *NA*, not applicable.

^a, b, c^No significant difference between groups containing the same letter (*p* < 0.05, Mann–Whitney U test/Tukey *post-hoc* test).

† Data is expressed as mean ± SD of fasting serums.

‡ Reference ranges were in accordance of publications of American Diabetes Association^19^, American Heart Association^11^ and Hacettepe University Hospital Biochemistry Laboratory.

Comparisons between strength athletes, endurance athletes and non-athletes analysed with Kruskal–Wallis test or one-way ANOVA test. *p* < 0.05 is significant.

Pairwise Mann–Whitney tests were adjusted for multiple comparisons within each variable using the Holm–Bonferroni method. Cells remaining significant after false discovery rate are shown in bold.

Among lipid parameters with reference ranges, only weightlifters in strength athletes showed low HDL-C, high LDL-C, and low ApoA-1 relative to reference values. All other subgroups had lipid profiles within normal limits. There were no significant differences among the three groups for TC or Lp(a) (*p* > 0.05). Several other parameters differed between athletes and non-athletes and between endurance and strength athletes (Table 3).

Endurance (87.4 ± 51.4 mg/dL) and strength athletes (93.1 ± 36.0 mg/dL) had lower TG than non-athletes (125.8 ± 58.7 mg/dL; *p* = 0.006). Similarly, endurance (17.5 ± 10.3 mg/dL) and strength athletes (18.6 ± 7.2 mg/dL) had lower VLDL-C levels than the non-athletes (23.0 ± 11.6 mg/dL; *p* = 0.031). TG and VLDL-C did not differ between endurance and strength athletes (*p* > 0.05) (Table 3).

Strength athletes (127.2 ± 29.0 mg/dL and 99.2 ± 27.1 mg/dL, respectively) had higher LDL-C and ApoB levels than endurance athletes (92.0 ± 16.0 mg/dL; *p* < 0.001 and 71.8 ± 10.6; *p* < 0.001, respectively) and non-athletes (105.9 ± 26.5 mg/dL; *p* = 0.022 and 86.4 ± 20.0 mg/dL; *p* > 0.05, respectively). Endurance athletes had lower ApoB levels than non-athletes (*p* < 0.05), whereas LDL-C did not differ significantly between these two groups (*p* > 0.05) (Table 3).

HDL-C and ApoA-1 were lower in strength athletes (38.8 ± 18.1 mg/dL and 102.0 ± 33.6 mg/dL respectively) than in endurance athletes (54.0 ± 9.7 mg/dL; *p* = 0.001 and 140.6 ± 18.7 mg/dL; *p* < 0.001, respectively). Endurance athletes also had higher HDL-C and ApoA-1 than non-athletes (47.5 ± 8.7 mg/dL; *p* = 0.001 and 121.8 ± 16.6 mg/dL; *p* < 0.001, respectively) (Table 3).

TC/HDL-C, LDL-C/HDL-C, and ApoB/ApoA-1 were higher in strength athletes (6.4 ± 4.6; 4.7 ± 4.0; 1.26 ± 1.1) than in endurance athletes (3.1 ± 0.6; 1.7 ± 0.4; 0.52 ± 0.1) (*p* < 0.001). Endurance athletes had lower LDL-C/HDL-C and ApoB/ApoA-1 than non-athletes (2.3 ± 0.7; 0.71 ± 0.2; *p* < 0.05) (Table 3).

Strength athletes (0.41 ± 0.3) had higher atherogenic index values than endurance athletes (0.16 ± 0.2; *p* = 0.004) but did not differ from non-athletes (0.40 ± 0.2; *p* > 0.05). Endurance athletes had lower atherogenic index values than non-athletes (*p* = 0.003) (Table 3).

Several lipid and lipoprotein markers differed across groups in the proportions below/above clinical cut-offs (Table 4). Non-athletes had the highest prevalence of hypertriglyceridemia (26.3%; *p* = 0.029), elevated Lp(a) (31.6%; *p* = 0.002), and high atherogenic index (84.2%; *p* = 0.006). In contrast, strength athletes had the highest prevalence of low HDL-C (48.9%; *p* = 0.001) and low ApoA-I (57.8%; *p* < 0.001), along with high LDL-C (37.8%; *p* = 0.003), high ApoB (44.4%; *p* = 0.001), elevated TC/HDL-C (42.2%; *p* = 0.001), LDL-C/HDL-C (51.1%; *p* < 0.001), and ApoB/ApoA-I (48.9%; *p* < 0.001). Differences in TC and VLDL-C were not significant. The proportion with ≥1 abnormal lipid marker (TC, TG, LDL-C, HDL-C) differed across groups (*p* < 0.001); 42.1% (*n* = 8) in non-athletes, 66.7% (*n* = 30) in strength athletes, and 15.0% (*n* = 3) in endurance athletes. No endurance athlete exceeded cut-offs for LDL-C, ApoA-1, ApoB, TC/HDL-C, LDL-C/HDL-C or ApoB/ApoA-1 (Table 4).

**Table 4:** Frequencies of athletes and non-athletes at risk for dyslipidemia.

Parameters^†^	Cut-off values^‡^	Strength athletes			Endurance athletes			Non-athletes (*n* = 19)	*p*
		Wrestlers (*n* = 29)	Weightlifters (*n* = 16)	Total (*n* = 45)	Runners (*n* = 15)	Cyclists (*n* = 5)	Total (*n* = 20)		
TC	≥200	8 (27.6)	4 (25.0)	12 (26.7)	1 (6.7)	–	1 (5.0)	4 (21.1)	0.098
TG	≥150	1 (3.4)	1 (6.4)	2 (4.4)	2 (13.3)	–	2 (10.0)	5 (26.3)	**0.029**
HDL-C	<40	7 (24.1)	15 (93.8)	22 (48.9)	1 (6.7)	–	1 (5.0)	4 (21.1)	**0.001**
LDL-C	≥130	8 (27.6)	9 (56.3)	17 (37.8)	–	–	–	4 (21.1)	**0.003**
VLDL-C	≥40	1 (3.4)	1 (6.3)	2 (4.4)	2 (13.3)	–	2 (10.0)	1 (5.3)	0.826
ApoA-1	<115	11 (37.9)	15 (93.8)	26 (57.8)	–	–	–	6 (31.6)	***p* < 0.001**
ApoB	>100	5 (17.2)	15 (93.8)	20 (44.4)	–	–	–	6 (31.6)	**0.001**
Lipoprotein (a)	>30	1 (3.4)	–	1 (2.2)	–	1 (20.0)	1 (5.0)	6 (31.6)	**0.002**
TC/HDL-C	>5.0	4 (13.8)	15 (93.8)	19 (42.2)	–	–	–	3 (15.8)	**0.001**
LDL-C/HDL-C	>3.0	8 (27.6)	15 (93.8)	23 (51.1)	–	–	–	4 (21.1)	***p* < 0.001**
ApoB/ApoA-1	≥0.90	7 (24.1)	15 (93.8)	22 (48.9)	–	–	–	4 (21.1)	***p* < 0.001**
Atherogenic index	>0.21	14 (48.3)	16 (100.0)	30 (66.7)	6 (40.0)	1 (20.0)	7 (35.0)	16 (84.2)	**0.006**
*Abnormal marker count* ^§^	*n ≥ 1*	14 (48.3)	16 (100.0)	30 (66.7)	3 (20.0)	–	3 (15.0)	8 (42.1)	***p* < 0.001**

**Notes:**

TC, total cholesterol; TG, triglycerides; HDL-C, high-density lipoprotein cholesterol; LDL-C, low-density lipoprotein cholesterol; VLDL-C, very low-density lipoprotein cholesterol; ApoA-1, Apolipoprotein A-1; ApoB, Apolipoprotein B.

‡ Cut-off values were used: TC ≥ 200 mg/dL, TG ≥ 150 mg/dL, HDL-C < 40 mg/dL (men), LDL-C ≥ 130 mg/dL, VLDL-C ≥ 40 mg/dL (Grundy et al., 2004), ApoA-1 < 115 mg/dL (Walldius & Jungner, 2004), ApoB > 100 mg/dL (Walldius & Jungner, 2004), Lp(a) >30 mg/dL (Kostner et al., 1981), TC/HDL-C > 5.0, LDL-C/HDL-C > 3.0 (Kubalová et al., 2025; Salcedo-Cifuentes et al., 2020), ApoB/ApoA-1 ≥ 0.90 (Walldius & Jungner, 2006), and Atherogenic Index >0.21 (Dobiášová et al., 2011).

† Lipids TC, TG, LDL-C, and HDL-C were used for abnormal marker count.

§ Values are *n* (%) of participants above/below cut-offs.

P: Fisher’s exact test (Monte-Carlo) for 2 × 3 tables when any expected cell <5; otherwise Pearson χ^2^. Cells remaining significant after false discovery rate are shown in bold.

### Relationship between the lipid profile, anthropometric measurements, and dietary intakes

The relationship between anthropometric measurements, dietary intakes, and lipid parameters was estimated with Pearson and Spearman Correlation analyses for both endurance and strength athletes (Tables S1, S2). Within each subgroup, a small set of correlations were significant. After BH-FDR control within-table (q < 0.05), surviving associations were shown in bold in Tables S1, S2; all others are exploratory. In endurance athletes, body fat (%, skinfold) was correlated positively with Lp(a) (r = 0.528, *p* = 0.029). There was also a positive correlation between body fat (%, BIA) and TC/HDL-C ratio (r = 0.459, *p* < 0.049). WHtR was positively correlated with LDL-C (r = 0.557, *p* = 0.016) and ApoB (r = 0.49, *p* = 0.039). Daily dietary energy intake correlated positively with ApoB (r = 0.543, *p* = 0.045) and TC/HDL-C ratio (r = 0.534, *p* = 0.049) in endurance athletes. However, no correlations remained significant after Benjamini-Hochberg FDR adjustment (Table S1).

In strength athletes, body fat (%, BIA) correlated positively with TG (r = 0.424, *p* = 0.004). Dietary energy intake correlated positively with TG (r = 0.559, *p* = 0.001), VLDL-C (r = 0.559, *p* = 0.001), and atherogenic index (r = 0.508, *p* = 0.002) and was negatively correlated with HDL-C (r = −0.399, *p* = 0.018). Dietary protein intake, expressed as g/kg, was positively correlated with TG (r = 0.426, *p* = 0.005), VLDL-C (r = 0.435, *p* = 0.005), TC/HDL-C (r = 0.414, *p* = 0.008), LDL-C/HDL-C (r = 0.419, *p* = 0.007), atherogenic index (r = 0.505, *p* = 0.001), and negatively correlated with HDL-C (r = −0.451, *p* = 0.003). Similarly, dietary fat intake was positively correlated with TG (r = 0.401, *p* = 0.009), ApoB (r = 0.426, *p* = 0.006), TC/HDL-C (r = 0.422, *p* = 0.007), LDL-C/HDL-C (r = 0.410, *p* = 0.009), ApoB/ApoA-1 (r = 0.419, *p* = 0.006), atherogenic index (r = 0.499, *p* = 0.001), and negatively correlated with HDL-C (r = −0.425, *p* = 0.006). Moreover, dietary carbohydrate intake was positively correlated with TG (r = 0.475, *p* = 0.002), VLDL-C (r = 0.463, *p* = 0.003), and atherogenic index (r = 0.462, *p* = 0.002). All the mentioned correlations in strength athletes were found significant after BH FDR adjustment. Effect sizes were classified as small (r = 0.10–0.29), moderate (r = 0.30–0.49) and large (r ≥ 0.50) (Table S2).

We tested four binary logistic regression models (main, sensitivity, control, and subgroup) for lipid outcomes. Our main model (A) was to test all variables (energy, CHO:PRO, fiber, WHtR, and study group) in our full sample. Model B (sensitivity model) replaced WHtR with body fat % to compare adiposity markers as predictors of dyslipidemia risk. Model C (control model) omitted adiposity to evaluate potential mediation by adiposity. Model D (subgroup model) applied the main specification to athletes only. Across models, multicollinearity was low **(**VIF = 1.017–2.949; tolerance = 0.339–0.983). Model fit was acceptable (Omnibus χ^2^ *p* < 0.05; Hosmer-Lemeshow *p* = 0.088–0.510; Nagelkerke R^2^ = 0.121–0.564).

**Figure 2:**
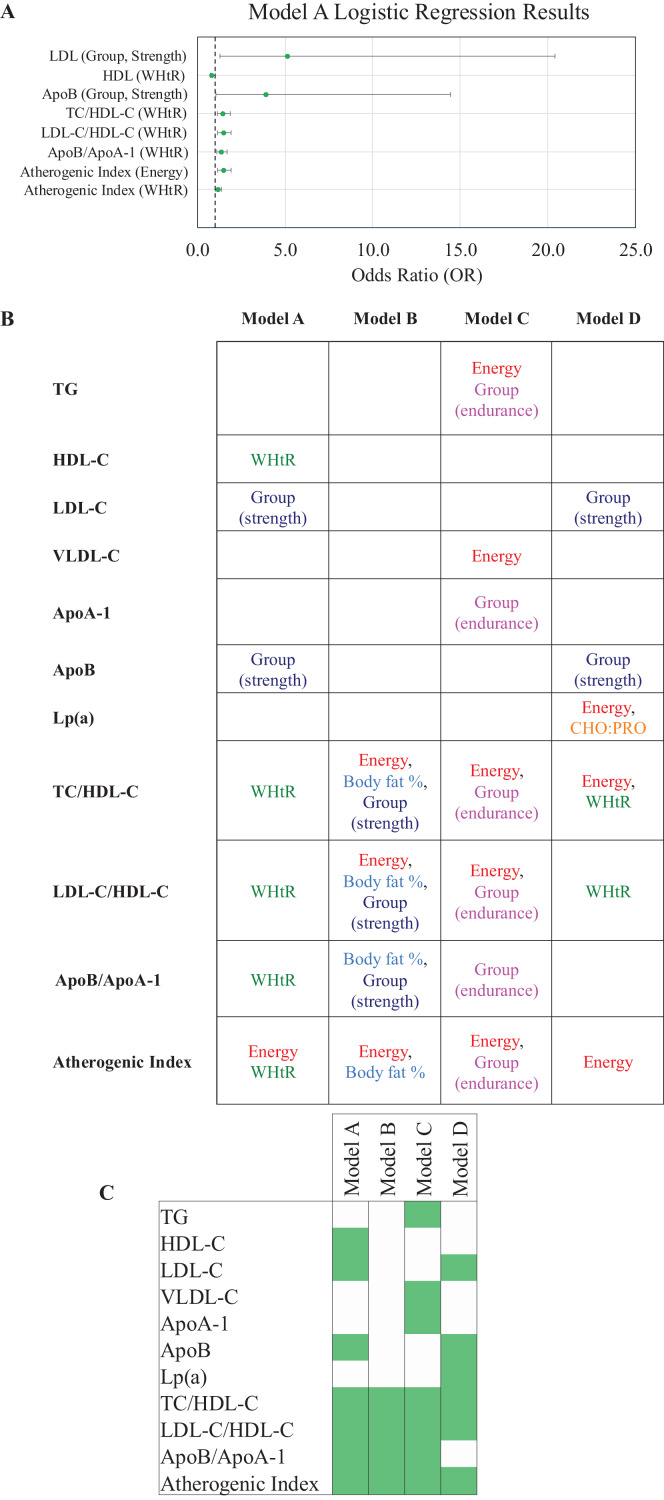
Binary logistic regression models and results. (A) Model A logistic regression results. (B) Table of significant predictors of lipid outcomes in logistic regression models A–D. (C) Heatmap of significant lipid outcomes in logistic regression models A–D. TC, total cholesterol; TG, triglycerides; HDL-C, high-density lipoprotein cholesterol; LDL-C, low-density lipoprotein cholesterol; VLDL-C, very low-density lipoprotein cholesterol; ApoA-1, Apolipoprotein A-1; ApoB, Apolipoprotein B; Lp(a), lipoprotein (a); WHtR, waist-to-height ratio, CHO:PRO[b], carbohydrate protein ratio.

In the main model (Model A) of this study strength athletes had significantly higher odds of elevated LDL-C (OR = 5.16, 95% CI [1.30–20.40], *p* = 0.019) and ApoB (OR = 3.91, 95% CI [1.06–14.44], *p* = 0.041) compared to non-athletes. Higher WHtR was a strong predictor of lower HDL-C (OR = 0.85, 95% CI [0.73–0.99], *p* = 0.042) and increased lipid ratios TC/HDL-C (OR = 1.455, 95% CI [1,143–1,852], *p* = 0.002), LDL-C/HDL-C (OR = 1.492, 95% CI [1,156–1,925], *p* = 0.002), and ApoB/ApoA-1 (OR= 1.352, 95% CI [1.094–1.67], *p* = 0.005). Additionally, WHtR (OR = 1.19, 95% CI [1.01–1,393], *p* = 0.038) and total energy intake (*p* = 0.008) were independently associated with higher atherogenic index (Fig. 2A, Table S3).

In Model B, where WHtR was replaced with body fat %, similar to Model A, body fat % was a significant predictor of elevated TC/HDL-C (OR = 1.22, 95% CI [1.06–1.40], *p* = 0.005), LDL-C/HDL-C (OR = 1.23, 95% CI [1.06–1.41], *p* = 0.006), and ApoB/ApoA-1 ratio (OR = 1.17, 95% CI [1.03–1.33], *p* = 0.017), as well as a higher atherogenic index (OR = 1.19, 95% CI [1.04–1.37], *p* = 0.013). Strength athletes remained more likely to present with elevated LDL-C (OR = 4.19, *p* = 0.051). Energy intake emerged as a consistent predictor of lipid ratios and atherogenic index (Figs. 2B, 2C, Table 3).

In Model C, where body composition was excluded, endurance athletes had significantly lower odds of elevated TG (OR = 0.09, 95% CI [0.02–0.53], *p* = 0.008), TC/HDL-C (OR = 0.057, 95% CI [0.01–0.53], *p* = 0.012), LDL-C/HDL-C (OR = 0.037, 95% CI [0.003–0.46], *p* = 0.010), ApoB/ApoA-1 (OR = 0.090, 95% CI [0.011–0.72], *p* = 0.010), and atherogenic index (OR = 0.011, 95% CI [0.001–0.16], *p* = 0.001), indicating a favorable lipid profile. ApoA-1 levels were significantly higher in endurance athletes (OR = 5.69, 95% CI [1.10–29.49], *p* = 0.038). Energy remained significant for TG, VLDL-C, lipid ratios, and atherogenic index (Figs. 2B, 2C, Table S3).

Model D (athletes only) confirmed that strength athletes had significantly greater odds of elevated LDL-C (OR = 17.42, *p* = 0.016) and ApoB (OR = 38.21, *p* = 0.004). Additionally, higher dietary energy intake was associated with increased odds of elevated Lp(a) (OR = 1.000, *p* = 0.045), and a higher CHO:PRO ratio was associated with lower odds of elevated Lp(a) (OR = 0.527, *p* = 0.044). Moreover, WHtR remained a significant predictor of adverse lipid ratios TC/HDL-C (OR = 1.275, *p* = 0.049) and LDL-C/HDL-C (OR = 1.306, *p* = 0.039) (Figs. 2B, 2C, Table S3).

An additional regression set excluded total energy and entered macronutrient intakes (g/kg) separately (Table S5). The findings remained consistent with the main analysis, showing that macronutrient intakes normalized by body weight followed the same directional trends as energy intake, and the overall interpretation was unchanged.

As a sensitivity analysis substituting total energy with macronutrient percent energy (protein, fat, carbohydrate) and adjusting for WHtR and study group, no macronutrient variable was independently associated with lipid outcomes, whereas WHtR remained significant across models. These findings indicate that adiposity is the principal predictor in our dataset rather than energy intake across macronutrients (Table S5).

As an additional sensitivity analysis, we applied the residual method (Willett, Howe & Kushi, 1997) to energy-adjust macronutrient intakes and re-ran Model A. Small nominal associations appeared for some residuals, but none remained significant after BH FDR correction. The effects of WHtR, study group, and energy were unchanged, except for the WHtR-HDL-C associations. Overall, these results again confirm that adiposity and total energy intake drive the lipid outcomes in this cohort (Table S5).

## Discussion

This study provides unique insights into the nutritional and physiological status of elite athletes in Türkiye. To our knowledge, no published data currently explore the relationship between dietary patterns, body composition and lipid profiles in elite Turkish athletes. We also applied binary logistic regression to identify independent predictors of lipid abnormalities, offering clinical perspectives on cardiovascular risk markers in athletes.

Endurance athletes had lower body weight, body fat, waist circumference and fat-free mass than strength athletes and non-athletes. Similar findings have been reported in Turkish male athletes (national- and Olympic-level athletes) (Balci et al., 2021) and participants who completed a 12-week endurance training program (Guo et al., 2011). In parallel, another study showed lower fat mass in sprint and endurance athletes compared to strength athletes and controls (Walker et al., 2023). Interestingly, body fat among wrestlers from our study was higher than that reported for Olympic wrestlers in Türkiye (Devrim-Lanpir et al., 2021). The discrepancy might be attributed to differences in study populations or variation in fat-free mass measurement methods (Guo et al., 2011). Consequently, the lower waist circumference, WHR, and WHtR in endurance athletes further support the association between aerobic exercise and healthier fat distribution (Jayedi et al., 2024). These cross-sectional associations likely reflect both training exposure and sport context.

Variations in fat distribution among athletes have important implications for blood lipid profiles and metabolic health. Although adiposity measures such as waist circumference, WHR, and WHtR are not routinely used in athletes, our study provides valuable data reinforcing their clinical relevance for blood lipid profiling. Higher body fat was associated with higher TG levels in strength athletes. Notably, our logistic regression models indicate that WHtR is a consistent predictor of adverse lipid outcomes, highlighting its potential use in cardiovascular risk assessment, even in athletes.

Our data demonstrate that endurance athletes engaged in aerobic physical activity exhibit low levels of TG, VLDL-C, LDL-C, ApoB, alongside high levels of HDL-C and ApoA-1, consistent with findings reported by others (Lira et al., 2010). In contrast, strength athletes (wrestlers and weightlifters) displayed higher TC/HDL-C and LDL-C/HDL-C ratios compared to endurance athletes (runners and cyclists). Beyond mean differences, group prevalence of dyslipidemia also diverged-non-athletes were highest for TG/Lp(a)/atherogenic index, strength athletes highest for low HDL-C/ApoA-I and elevated lipid ratios, while endurance athletes had no cases above cut-offs for LDL-C, ApoA-I, ApoB, or lipid ratios. Because the strength cohort competes in weight-class sports, between-group differences may reflect sport-specific behaviors (*e.g*., rapid weight loss/weight cycling) in addition to training characteristics (Franchini, Brito & Artioli, 2012; Marten et al., 2025). Although aerobic and anaerobic training have been proposed to differentially affect lipid metabolism and lipolytic activity (Guo et al., 2011; Petridou & Mougios, 2022), a randomized cross-over study indicates that average lipid changes do not differ meaningfully between endurance and resistance training, with considerable inter-individual heterogeneity (Thomas et al., 2022). It is also important to note that the low levels of HDL-C observed in strength athletes might be influenced by individual genetic background (Hodoglugil, Williamson & Mahley, 2010), specific polymorphisms (Spielmann et al., 2007) or lifestyle factors unrelated to exercise (Petridou, Lazaridou & Mougios, 2005). Furthermore, it has been reported that Turkish people have strikingly low levels of HDL-C (10–15 mg/dL lower than those of Americans or Western Europeans) associated with elevated hepatic lipase mass and activity (Hodoglugil, Williamson & Mahley, 2010). Additionally, Lp(a) levels are predominantly genetically determined, so inter-individual differences in Lp(a) among athletes largely reflect inherited biology rather than training or diet (Reyes-Soffer et al., 2022).

The well-known relationship between exercise metabolism and blood lipid-lipoprotein profiles underscores the importance of the dietary patterns of athletes (Rodriguez, DiMarco & Langley, 2009; Thomas, Erdman & Burke, 2016; Zalcman et al., 2007). Excessive protein and fat intake has been reported in athletes (Zalcman et al., 2007), and higher dietary fat intake can displace carbohydrate and reduce muscle and liver glycogen storage, which is crucial for optimal performance. In our study, weightlifters within the strength cohort showed slightly increased fat intake (% of energy), which may contribute to boosting intramuscular triglyceride stores (Juzwiak et al., 2008; Thomas, Erdman & Burke, 2016). Nonetheless, prolonged high-fat diets may lead to adverse health outcomes, (Juzwiak et al., 2008) as a diet rich in saturated fats and cholesterol are risk factors (Mansour et al., 2024). Similarly, dietary patterns high in saturated fat and cholesterol, such as ketogenic or high-fat diets, have been associated with elevated LDL-C and TC levels in athletes, as noted by Creighton et al. (2018) in keto-adapted ultra-endurance runners.

At moderate exercise intensities, fat oxidation contributes substantially to energy production, whereas at higher intensities carbohydrate metabolism becomes the predominant energy source (Thomas, Erdman & Burke, 2016). Consistent with this, endurance athletes in this study exhibited a higher CHO:PRO ratio, reflecting their increased reliance on carbohydrates as a primary energy source; a CHO:PRO ratio of 3:1–4:1 may promote glycogen resynthesis and optimize performance (Kerksick et al., 2008). The CHO:PRO ratio reported here reflects habitual dietary intake patterns and should not be interpreted as a direct indicator of substrate utilization during exercise, which would require metabolic measurements such as respiratory exchange ratio *via* indirect calorimetry. In summary, current evidence does not support either a high-fat/low-carbohydrate diet or very low-fat intake (<20% of energy) for optimal performance or long-term health (Thomas, Erdman & Burke, 2016), and carbohydrate availability can modulate endurance training adaptations (Gejl et al., 2017), providing a physiological rationale for the more favorable lipid profile observed in our endurance group.

Importantly, our regression models demonstrated that dietary energy intake, regardless of macronutrient composition, may negatively affect the lipid profile even after adjusting for adiposity. However, endurance athletes consumed the highest energy and macronutrient intakes (g/kg) yet exhibited favorable lipid profiles and lower WHtR, whereas strength athletes had lower reported energy intake but higher WHtR and more frequent dyslipidemia. The higher energy intake and healthier lipid profile in endurance athletes suggest that excess energy consumption, rather than total energy intake *per se*, mediates the development in adiposity which may in turn lead to a high-risk lipid profile associated with WHtR. This may also be supported by the positive correlations of energy, protein, fat, and carbohydrate intake with higher TG, VLDL-C, and lipid ratios, and lower HDL-C in strength athletes. Although endurance athletes reported higher macronutrient intakes (g/kg), their dietary fat as a percentage of energy was lower than in strength athletes which may also favor lipid profiles and lower WHtR. Overall, meeting sport-specific carbohydrate needs (Kerksick et al., 2008) aligns with a healthier lipid profile, whereas excess energy that promotes adiposity may blunt these benefits, especially in weight-class sports.

The lipid abnormalities observed in our cohort, particularly in strength athletes, may have long-term implications for cardiovascular health. Similar patterns have been reported in other athletic populations. For example, a recent study involving acrobatic and tumbling athletes-also classified as strength athletes-identified dyslipidemia in a portion of their sample, despite their competitive training status (de Souza et al., 2024). Moreover, a large Olympic cohort reported more favorable lipids in Afro-Caribbean *vs*. Caucasian athletes, and better profiles in endurance *vs*. non-endurance within Caucasians, emphasizing ethnic/sex/sport-discipline effects beyond training volume alone (Di Gioia et al., 2024). In soccer (endurance-dominant aerobic load and repeated anaerobic bursts), seasonal data were associated with higher HDL and lower TC/HDL ratios *vs*. non-athletes (Marsigliante et al., 2025). Broadly, professional athletes similarly note lower TC/LDL/TG and equal or higher HDL *vs*. sedentary peers, while highlighting modifying roles of diet, origin, training regimen, and genetics (Agostinete et al., 2017; Varaeva et al., 2020). These findings suggest that certain sport-specific training and dietary practices may predispose athletes to a less favorable lipid profile, supporting the importance of tailored nutritional interventions within different sport disciplines.

Our study has potential limitations to consider. As an observational study, it did not include off-season training lipid profiles, though it is uncommon for elite athletes to experience extended periods without training. Additionally, we did not quantify training load (frequency, duration, intensity) which may influence energy and macronutrient intake as well as lipid metabolism. Although all athletes were actively training at the time of data collection, they were not in the competition phase. We did not measure rapid weight loss/weight cycling common in weight-class sports, which may be associated with their unfavorable lipid profile (Maksimovic et al., 2024). Additionally, other dietary factors, such as cholesterol intake, fatty acid profile, fiber type, antioxidant capacity, and micronutrient intake of the diets consumed were not analyzed, which are known to influence blood lipid profiles. Future studies should investigate these limitations for a more comprehensive analysis.

## Conclusions

Regular physical activity can improve blood lipid profiles; nonetheless, athletes may still experience dyslipidemia. In this cohort, endurance athletes exhibited a lipid profile generally considered protective against atherosclerosis, whereas strength athletes showed a less favorable pattern. These differences appear to relate more to adiposity and diet than to training modality. Adiposity markers-particularly waist-to-height ratio and body fat percentage-were the most consistent independent predictors of adverse lipid outcomes, and higher dietary energy was associated with a more atherogenic profile. Notably, endurance athletes reported the highest energy and macronutrient intakes (g/kg) yet had lower adiposity and healthier blood lipids, underscoring that excess energy (relative to requirements), rather than total energy, is the likely driver of risk *via* greater adiposity. This pattern is likely attributable to the higher training volume and associated energy requirements of endurance athletes. Our findings are mostly in agreement with reports from other regions showing that athletes’ lipid profiles vary with population genetics (*e.g*., HDL-C, Lp(a) levels) and environmental factors (*e.g*., smoking). Overall, ensuring adequate carbohydrate to meet sport demands while avoiding energy excess that promotes adiposity may support healthier lipid-lipoprotein profiles in competitive athletes. Collectively, these cross-sectional findings indicate that lipid-lipoprotein profiles differ across athlete groups and relate more consistently to adiposity and diet, underscoring the importance of sport-specific nutrition to enhance health, physical performance, and early recognition of health abnormalities.

## Supplemental Information

10.7717/peerj.20743/supp-1 Supplemental Information 1 Demographics.

10.7717/peerj.20743/supp-2 Supplemental Information 2 Supplementary Tables.

10.7717/peerj.20743/supp-3 Supplemental Information 3 Raw data.

## Abbreviations

ApoA-1: Apolipoprotein A-1
ApoB: Apolipoprotein B
BIA: multi-frequency bioelectrical impedance analyzer
BMI: body mass index
HDL-C: high-density lipoprotein cholesterol
LDL-C: low-density lipoprotein cholesterol
Lp(a): lipoprotein (a)
TC: total cholesterol
TG: triglycerides
VLDL-C: very low-density lipoprotein cholesterol
WHR: waist-to-hip ratio
WHtR: waist-to-height ratio

## Additional Information and Declarations

### Data Availability

The following information was supplied regarding data availability:

The raw data is available in the Supplemental File.
