# Supplementary material for: Waist-to-height ratio, body fat, and macronutrient intake as predictors of lipid abnormalities in elite Turkish athletes: a comparative study
Source: PeerJ. 2026 Feb 10;14:e20743. doi: 10.7717/peerj.20743 (PMC12903904; doi:10.7717/peerj.20743)
Supplement: Supplemental Information 2 [file peerj-14-20743-s002.docx]

**Supplementary Table 1**. Correlation of the lipid profile, anthropometric measurements and dietary intakes in endurance athletes

| **Parameters** | Body Fat  (%, skinfold) | | Body Fat  (%, BIA) | | WHtR | | Energy (kcal/day) | | Protein  (g/kg) | | Fat  (g/kg) | | | Carbohydrate (g/kg) | | | CHO:PRO | | Fiber (g/day) | | |
| --- | --- | --- | --- | --- | --- | --- | --- | --- | --- | --- | --- | --- | --- | --- | --- | --- | --- | --- | --- | --- | --- |
|  | r | p | r | p | r | p | r | p | r | p | | r | p | | r | p | r | p | | r | p |
| TC | -0.093 | 0.722 | 0.217 | 0.357 | 0.390 | 0.110 | 0.277 | 0.337 | 0.340 | 0.168 | | 0.368 | 0.133 | | 0.330 | 0.182 | 0.159 | 0.503 | | 0.210 | 0.374 |
| TG | -0.223 | 0.390 | -0.014 | 0.955 | 0.002 | 0.994 | 0.319 | 0.267 | 0.327 | 0.185 | | 0.174 | 0.485 | | 0.294 | 0.236 | 0.031 | 0.895 | | 0.099 | 0.677 |
| HDL-C | 0.183 | 0.482 | 0.434 | 0.056 | -0.012 | 0.963 | -0.123 | 0.674 | -0.131 | 0.603 | | -0.060 | 0.814 | | -0.081 | 0.750 | 0.044 | 0.854 | | 0.030 | 0.900 |
| LDL-C | -0.256 | 0.321 | -0.004 | 0.987 | 0.557^§^ | 0.016 | 0.304 | 0.291 | 0.360 | 0.142 | | 0.440 | 0.067 | | 0.377 | 0.123 | 0.176 | 0.458 | | 0.311 | 0.181 |
| VLDL-C | -0.223 | 0.390 | -0.14 | 0.955 | 0.002 | 0.994 | 0.319 | 0.267 | 0.327 | 0.185 | | 0.176 | 0.485 | | 0.294 | 0.236 | 0.031 | 0.895 | | 0.099 | 0.677 |
| ApoA-1 | -0.162 | 0.535 | 0.202 | 0.394 | 0.058 | 0.820 | 0.226 | 0.436 | 0.501^§^ | 0.034 | | 0.492^‡^ | 0.038 | | 0.509^§^ | 0.031 | 0.162 | 0.495 | | 0.389 | 0.090 |
| ApoB | -0.135 | 0.606 | 0.104 | 0.663 | 0.490^‡^ | 0.039 | 0.543^§^ | 0.045 | 0.401 | 0.099 | | 0.336 | 0.173 | | 0.348 | 0.157 | 0.159 | 0.503 | | 0.236 | 0.316 |
| Lipoprotein (a) | 0.528^§^ | 0.029 | 0.135 | 0.571 | -0.255 | 0.307 | -0.085 | 0.774 | -0.227 | 0.366 | | -0.240 | 0.337 | | -0.208 | 0.408 | 0.031 | 0.895 | | -0.407 | 0.075 |
| TC/HDL-C | -0.392 | 0.119 | 0.459^‡^ | 0.049 | 0.208 | 0.409 | 0.534^§^ | 0.049 | 0.375 | 0.126 | | 0.270 | 0.278 | | 0.358 | 0.145 | 0.044 | 0.854 | | 0.144 | 0.544 |
| LDL-C/HDL-C | -0.397 | 0.115 | -0.412 | 0.071 | 0.278 | 0.264 | 0.530 | 0.051 | 0.383 | 0.117 | | 0.334 | 0.176 | | 0.375 | 0.126 | 0.176 | 0.458 | | 0.227 | 0.336 |
| ApoB/ApoA1 | -0.078 | 0.757 | -0.057 | 0.823 | 0.305 | 0.219 | -0.029 | 0.905 | -0.121 | 0.633 | | -0.137 | 0.588 | | -0.152 | 0.548 | -0.107 | 0.654 | | -0.165 | 0.486 |
| Atherogenic Index | -0.299 | 0.244 | -0.229 | 0.332 | 0.049 | 0.848 | 0.389 | 0.169 | 0.286 | 0.250 | | 0.224 | 0.372 | | 0.271 | 0.276 | 0.031 | 0.895 | | 0.030 | 0.900 |

BIA, bioelectrical impedance analysis; WHtR, waist-to-height ratio; CHO:PRO, carbohydrate:protein ratio; TC, total cholesterol; TG, triglycerides; HDL-C, high-density lipoprotein cholesterol; LDL-C, low-density lipoprotein cholesterol; VLDL-C, very low-density lipoprotein cholesterol; ApoA-1, Apolipoprotein A-1; ApoB, Apolipoprotein B.

r, Pearson/Spearman Correlation Coefficient, p<0.05 is significant. Magnitude codes: ^†^ small, ^‡^ moderate, ^§^ large.

No correlations remained significant after Benjamini-Hochberg FDR adjustment; cells show raw p-values.

**Supplementary Table 2**. Correlation of the lipid profile, anthropometric measurements and dietary intakes in strength athletes

| **Parameters** | Body Fat  (%, skinfold) | | Body Fat  (%, BIA) | | WHtR | | Energy (kcal/day) | | | Protein  (g/kg) | | | Fat  (g/kg) | | Carbohydrate (g/kg) | | CHO:PRO | | | Fiber (g/day) | | |
| --- | --- | --- | --- | --- | --- | --- | --- | --- | --- | --- | --- | --- | --- | --- | --- | --- | --- | --- | --- | --- | --- | --- |
|  | r | p | r | p | r | p | | r | p | r | p | r | | p | r | p | r | p | r | | p |  |
| TC | 0.139 | 0.381 | 0.044 | 0.778 | -0.037 | 0.819 | | 0.075 | 0.675 | 0.074 | 0.650 | 0.108 | | 0.508 | 0.002 | 0.990 | 0.051 | 0.754 | -0.172 | | 0.282 |  |
| TG | 0.310^‡^ | 0.043 | **0.424**^‡^ | **0.004** | 0.292 | 0.061 | | **0.559**^§^ | **<0.001** | **0.426**^‡^ | **0.005** | **0.401**^‡^ | | **0.009** | **0.475**^‡^ | **0.002** | 0.079 | 0.619 | 0.087 | | 0.584 |  |
| HDL-C | -0.029 | 0.853 | -0.154 | 0.319 | -0.217 | 0.167 | | -0.399^‡^ | 0.018 | **-0.451**^‡^ | **0.003** | **-0.425**^‡^ | | **0.006** | -0.345^‡^ | 0.027 | 0.224 | 0.154 | -0.318^‡^ | | 0.040 |  |
| LDL-C | 0.115 | 0.462 | 0.069 | 0.657 | 0.056 | 0.729 | | 0.202 | 0.245 | 0.231 | 0.152 | 0.264 | | 0.100 | 0.137 | 0.400 | -0.105 | 0.508 | 0.009 | | 0.954 |  |
| VLDL-C | 0.310^‡^ | 0.043 | 0.424^‡^ | 0.004 | 0.312^‡^ | 0.047 | | **0.559**^§^ | **<0.001** | **0.435**^‡^ | **0.005** | 0.390^‡^ | | 0.013 | **0.463**^‡^ | **0.003** | 0.079 | 0.619 | 0.081 | | 0.615 |  |
| ApoA-1 | 0.001 | 0.997 | -0.092 | 0.553 | -0.196 | 0.214 | | -0.327 | 0.055 | -0.348^‡^ | 0.026 | -0.351^‡^ | | 0.024 | -0.247 | 0.119 | 0.203 | 0.197 | -0.321^‡^ | | 0.038 |  |
| ApoB | -0.018 | 0.907 | 0.102 | 0.510 | 0.035 | 0.828 | | 0.274 | 0.111 | 0.391^‡^ | 0.012 | **0.426**^‡^ | | **0.006** | 0.275 | 0.082 | -0.158 | 0.317 | 0.150 | | 0.344 |  |
| Lipoprotein (a) | -0.094 | 0.547 | -0.136 | 0.380 | -0.128 | 0.418 | | -0.093 | 0.596 | -0.299 | 0.057 | -0.292 | | 0.064 | -0.341^‡^ | 0.029 | 0.045 | 0.776 | -0.048 | | 0.763 |  |
| TC/HDL-C | 0.055 | 0.727 | 0.160 | 0.304 | 0.222 | 0.163 | | 0.420^‡^ | 0.013 | **0.414**^‡^ | **0.008** | **0.422**^‡^ | | **0.007** | 0.335^‡^ | 0.035 | -0.143 | 0.371 | 0.362^‡^ | | 0.020 |  |
| LDL-C/HDL-C | 0.048 | 0.762 | 0.147 | 0.348 | 0.222 | 0.164 | | 0.417^‡^ | 0.014 | **0.419**^‡^ | **0.007** | **0.410**^‡^ | | **0.009** | 0.311 | 0.051 | -0.159 | 0.320 | 0.364^‡^ | | 0.019 |  |
| ApoB/ApoA1 | -0.095 | 0.556 | 0.014 | 0.927 | 0.145 | 0.359 | | 0.405^‡^ | 0.007 | 0.392^‡^ | 0.011 | **0.419**^‡^ | | **0.006** | 0.279 | 0.078 | -0.235 | 0.134 | 0.170 | | 0.281 |  |
| Atherogenic Index | 0.134 | 0.391 | 0.275 | 0.071 | 0.235 | 0.135 | | **0.508**^§^ | **0.002** | **0.505**^§^ | **0.001** | **0.499**^‡^ | | **0.001** | **0.462**^‡^ | **0.002** | -0.086 | 0.586 | 0.325^‡^ | | 0.036 |  |

BIA, bioelectrical impedance analysis; WHtR, waist-to-height ratio; CHO:PRO, carbohydrate:protein ratio; TC, total cholesterol; TG, triglycerides; HDL-C, high-density lipoprotein cholesterol; LDL-C, low-density lipoprotein cholesterol; VLDL-C, very low-density lipoprotein cholesterol; ApoA-1, Apolipoprotein A-1; ApoB, Apolipoprotein B.

r, Pearson/Spearman Correlation Coefficient, p<0.05 is significant. Magnitude codes: ^†^ small, ^‡^ moderate, ^§^ large.

**Bold**: significant after Benjamini-Hochberg FDR adjusment (q<0.05; q range: 0.036-0.045) within table; cells show raw p-values.

**Supplementary Table 3.** Logistic regression analysis results for the lipid profile across Model A-D.

| **Model** | **Lipid Outcome** | **Variable** | **B** | **S.E.** | **Wald** | **df** | **p-value** | **OR (Exp(B))** | **95% CI Lower** | **95% CI Upper** |
| --- | --- | --- | --- | --- | --- | --- | --- | --- | --- | --- |
| A | LDL-C | Group |  |  | 10.374 | 2 | 0.006 |  |  |  |
|  |  | Group (strength) | 1.640 | 0.702 | 5.462 | 1 | 0.019 | 5.156 | 1.303 | 20.402 |
|  | HDL-C | WHtR | -0.163 | 0.80 | 4.136 | 1 | 0.042 | 0.849 | 0.726 | 0.994 |
|  | ApoB | Group |  |  | 11.055 | 2 | 0.004 |  |  |  |
|  |  | Group (strength) | 1.364 | 0.666 | 4.187 | 1 | 0.041 | 3.910 | 1.059 | 14.437 |
|  | TC/HDL-C | WHtR | 0.375 | 0.123 | 9.272 | 1 | 0.002 | 1.455 | 1.143 | 1.852 |
|  | LDL-C/HDL-C | WHtR | 0.400 | 0.130 | 9.428 | 1 | 0.002 | 1.492 | 1.156 | 1.925 |
|  | ApoB/ApoA1 | WHtR | 0.301 | 0.108 | 7.807 | 1 | 0.005 | 1.352 | 1.094 | 1.670 |
|  | Atherogenic Index | Energy | 0.000 | 0.000 | 6.957 | 1 | 0.008 | 1.000 | 1.000 | 1.001 |
|  |  | WHtR | 0.170 | 0.082 | 4.321 | 1 | 0.038 | 1.186 | 1.010 | 1.393 |
| B | LDL-C | Group |  |  | 11.292 | 2 | 0.004 |  |  |  |
|  |  | Group (strength) | 1.433 | .0735 | 3.804 | 1 | 0.051 | 4.192 | 0.993 | 17.701 |
|  | ApoB | Group |  |  | 9.833 | 2 | 0.007 |  |  |  |
|  | TC/HDL-C | Energy | 0.000 | 0.000 | 4.757 | 1 | 0.029 | 1.000 | 1.000 | 1.001 |
|  |  | Body fat % | 0.199 | 0.071 | 7.835 | 1 | 0.005 | 1.221 | 1.062 | 1.404 |
|  |  | Group |  |  | 7.343 | 2 | 0.025 |  |  |  |
|  |  | Group (strength) | 1.830 | 0.869 | 4.439 | 1 | 0.035 | 6.235 | 1.136 | 34.224 |
|  | LDL-C/HDL-C | Energy | 0.000 | 0.000 | 4.198 | 1 | 0.040 | 1.000 | 1.000 | 1.001 |
|  |  | Body fat % | 0.203 | 0.073 | 7.697 | 1 | 0.006 | 1.225 | 1.061 | 1.413 |
|  |  | Group |  |  | 7.978 | 2 | 0.019 |  |  |  |
|  |  | Group (strength) | 1.842 | 0.875 | 4.433 | 1 | 0.035 | 6.312 | 1.136 | 35.075 |
|  | ApoB/ApoA1 | Body fat % | 0.158 | 0.066 | 5.699 | 1 | 0.017 | 1.172 | 1.029 |  |
|  |  | Group |  |  | 7.312 | 2 | 0.026 |  |  |  |
|  |  | Group (strength) | 1.647 | 0.815 | 4.086 | 1 | 0.043 | 5.192 | 1.051 | 25.637 |
|  | Atherogenic Index | Energy | 0.000 | 0.000 | 8.077 | 1 | 0.004 | 1.000 | 1.000 | 1.001 |
|  |  | Body fat % | 0.175 | 0.071 | 6.120 | 1 | 0.013 | 1.191 | 1.037 | 1.369 |
| C | LDL-C | Group |  |  | 11.287 | 2 | 0.004 |  |  |  |
|  |  | Group (strength) | 1.156 | 0.605 | 3.645 | 1 | 0.056 | 3.176 | 0.970 | 10.404 |
|  | TG | Energy | 0.000 | 0.000 | 6.234 | 1 | 0.013 | 1.000 | 1.000 | 1.001 |
|  |  | Group |  |  | 7.203 | 2 | 0.027 |  |  |  |
|  |  | Group (endurance) | -2.399 | 0.898 | 7.134 | 1 | 0.008 | 0.091 | 0.016 | 0.528 |
|  | HDL-C | Group |  |  | 6.741 | 2 | 0.034 |  |  |  |
|  |  | Group (endurance) | 1.576 | 0.884 | 3.177 | 1 | 0.075 | 4.835 | 0.855 | 27.350 |
|  | VLDL-C | Energy | 0.000 | 0.000 | 5.844 | 1 | 0.016 | 1.000 | 1.000 | 1.001 |
|  | ApoA-1 | Group |  |  | 6.230 | 2 | 0.044 |  |  |  |
|  |  | Group (endurance) | 1.739 | 0.839 | 4.291 | 1 | 0.038 | 5.691 | 1.098 | 29.488 |
|  | ApoB | Group |  |  | 10.633 | 2 | 0.005 |  |  |  |
|  |  | Group (endurance) | -1.859 | 0.957 | 3.770 | 1 | 0.052 | 0.156 | 0.024 | 1.018 |
|  | TC/HDL-C | Energy | 0.000 | 0.000 | 4.922 | 1 | 0.027 | 1.000 | 1.000 | 1.001 |
|  |  | Group |  |  | 9.395 | 2 | 0.009 |  |  |  |
|  |  | Group (endurance) | -2.869 | 1.138 | 6.363 | 1 | 0.012 | 0.057 | 0.006 | 0.527 |
|  | LDL-C/HDL-C | Energy | 0.000 | 0.000 | 4.439 | 1 | 0.035 | 1.000 | 1.000 | 1.001 |
|  |  | Group |  |  | 9.258 | 2 | 0.010 |  |  |  |
|  |  | Group (endurance) | -3.294 | 1.285 | 6.574 | 1 | 0.010 | 0.037 | 0.003 | 0.460 |
|  | ApoB/ApoA1 | Group |  |  | 9.009 | 2 | 0.011 |  |  |  |
|  |  | Group (endurance) | -2.411 | 1.063 | 5.147 | 1 | 0.023 | 0.090 | 0.011 | 0.720 |
|  | Atherogenic Index | Energy | 0.001 | 0.000 | 7.701 | 1 | 0.006 | 1.001 | 1.000 | 1.001 |
|  |  | Group |  |  | 11.046 | 2 | 0.004 |  |  |  |
|  |  | Group (endurance) | -4.527 | 1.362 | 11.045 | 1 | 0.001 | 0.011 | 0.001 | 0.156 |
| D | LDL-C | Group (strength) | 2.858 | 1.183 | 5.834 | 1 | 0.016 | 17.424 | 1.714 | 177.104 |
|  | ApoB | Group (strength) | 3.643 | 1.267 | 8.269 | 1 | 0.004 | 38.206 | 3.190 | 457.582 |
|  | Lp(a) | Energy | 0.000 | 0.000 | 4.851 | 1 | 0.028 | 1.000 | 0.999 | 1.000 |
|  |  | CHO:PRO | -0.640 | 0.318 | 4.044 | 1 | 0.044 | 0.527 | 0.282 | 0.984 |
|  | TC/HDL-C | Energy | 0.000 | 0.000 | 4.013 | 1 | 0.045 | 1.000 | 1.000 | 1.001 |
|  |  | WHtR | 0.243 | 0.123 | 3.871 | 1 | 0.049 | 1.275 | 1.001 | 1.623 |
|  | LDL-C/HDL-C | WHtR | 0.267 | 0.129 | 4.241 | 1 | 0.039 | 1.306 | 1.013 | 1.683 |
|  | ApoB/ApoA1 | CHO:PRO | -0.762 | 0.415 | 3.377 | 1 | 0.066 | 0.467 | 0.207 | 1.052 |
|  | Atherogenic Index | Energy | 0.000 | 0.000 | 6.022 | 1 | 0.014 | 1.000 | 1.000 | 1.001 |

OR, odds ratio; LDL-C, low-density lipoprotein cholesterol; HDL-C, high-density lipoprotein cholesterol; WHtR, waist-to-height ratio; ApoB, Apolipoprotein B; TC, total cholesterol; TG, triglycerides; VLDL-C, very low-density lipoprotein cholesterol; ApoA-1, Apolipoprotein A-1; Lp(a), lipoprotein (a); CHO:PRO, carbohydrate:protein ratio.

Odds ratios (OR) and 95% confidence intervals (CI) are shown.

Models adjusted for energy, CHO:PRO, fiber, WHtR, body fat %, and study (strength, endurance, non-athletes).

After Benjamini–Hochberg FDR within models, conclusions were unchanged.

**Supplementary Table 4**. Comparison of correlation and logistic regression analysis results

| **Lipid Outcome** | **Correlation (Endurance Athletes)** | **Correlation**  **(Strength Athletes)** | **Logistic Regression Significant Predictor** |
| --- | --- | --- | --- |
| TC | ⚪ (no) | ⚪ (no) | ⚪ (no) |
| TG | ⚪ (no) | ✅ (Body fat, energy, protein, fat, carbohydrate positive) | ✅ (Energy positive) |
| HDL-C | ⚪ (no) | ❌ (Protein, fat negative) | ❌ (WHtR negative) |
| LDL-C | ⚪ (no) | ⚪ (no) | ✅ (Group strength positive) |
| VLDL-C | ⚪ (no) | ✅ (Energy, protein, carbohydrate positive) | ✅ (Energy positive) |
| ApoA-1 | ⚪ (no) | ⚪ (no) | ✅ (Group endurance positive) |
| ApoB | ⚪ (no) | ✅ (Fat positive) | ✅ (Group strength positive) |
| Lipoprotein (a) | ⚪ (no) | ⚪ (no) | ❌ (CHO:PRO negative) |
| TC/HDL-C | ⚪ (no) | ✅ (Protein, fat positive) | ✅ (WHtR, energy, body fat, group strength positive)  ❌ (Group endurance negative) |
| LDL-C/HDL-C | ⚪ (no) | ✅ (Protein, fat positive) | ✅ (WHtR, energy, body fat, group strength positive)  ❌ (Group endurance negative) |
| ApoB/ApoA1 | ⚪ (no) | ✅ (Fat, positive) | ✅ (WHtR, body fat, group strength positive)  ❌ (Group endurance negative) |
| Atherogenic Index | ⚪ (no) | ✅ (Energy, protein, fat, carbohydrate positive) | ✅ (Energy, WHtR, body fat positive)  ❌ (Group endurance negative) |

✅, Significant positive correlation/predictor; ❌, Significant negative correlation/predictor; ⚪, No significant association

TC, total cholesterol; TG, triglycerides; HDL-C, high-density lipoprotein cholesterol; WHtR, waist-to-height ratio; LDL-C, low-density lipoprotein cholesterol; VLDL-C, very low-density lipoprotein cholesterol; ApoA-1, Apolipoprotein A-1; ApoB, Apolipoprotein B; CHO:PRO, carbohydrate:protein ratio.

**Supplementary Table 5.** Logistic regression analysis results using macronutrient intakes (g/kg, % energy, and energy-adjusted residuals) for the lipid profile across Model A.

| **Model** | **Lipid Outcome** | **Variable** | **B** | **S.E.** | **Wald** | **df** | **p-value** | **OR (Exp(B))** | **95% CI Lower** | **95% CI Upper** |
| --- | --- | --- | --- | --- | --- | --- | --- | --- | --- | --- |
| Protein Intake (g/kg) | LDL-C | Group |  |  | 10.468 | 2 | 0.005 |  |  |  |
|  |  | Group (strength) | 1.621 | 0.692 | 5.485 | 1 | 0.019 | 5.056 | 1.303 | 19.624 |
|  | HDL-C | WHtR | -0.176 | 0.79 | 5.009 | 1 | 0.025 | 0.838 | 0.719 | 0.978 |
|  | ApoB | Group |  |  | 12.171 | 2 | 0.002 |  |  |  |
|  |  | Group (strength) | 1.366 | 0.661 | 4.276 | 1 | 0.039 | 3.921 | 1.074 | 14.317 |
|  | TC/HDL-C | WHtR | 0.400 | 0.123 | 10.584 | 1 | 0.001 | 1.491 | 1.172 | 1.897 |
|  | LDL-C/HDL-C | WHtR | 0.420 | 0.128 | 10.664 | 1 | 0.001 | 1.521 | 1.183 | 1.957 |
|  | ApoB/ApoA1 | WHtR | 0.292 | 0.101 | 8.429 | 1 | 0.004 | 1.340 | 1.100 | 1.632 |
|  | Atherogenic Index | Protein intake (g/kg) | 0.835 | 0.307 | 7.408 | 1 | 0.006 | 2.305 | 1.263 | 4.206 |
|  |  | WHtR | 0.216 | 0.084 | 6.576 | 1 | 0.010 | 1.241 | 1.052 | 1.463 |
| Protein Intake (% of energy) | LDL-C | Group |  |  | 10.541 | 2 | 0.005 |  |  |  |
|  |  | Group (strength) | 1.751 | 0.702 | 6.223 | 1 | 0.013 | 5.760 | 1.455 | 22.799 |
|  | HDL-C | WHtR | -0.167 | 0.079 | 4.389 | 1 | 0.036 | 0.847 | 0.724 | 0.989 |
|  | ApoB | Group |  |  | 11.271 | 2 | 0.004 |  |  |  |
|  |  | Group (strength) | 1.472 | 0.656 | 5.037 | 1 | 0.025 | 4.360 | 1.205 | 15.773 |
|  | TC/HDL-C | WHtR | 0.375 | 0.120 | 9.702 | 1 | 0.002 | 1.455 | 1.149 | 1.843 |
|  | LDL-C/HDL-C | WHtR | 0.400 | 0.127 | 9.880 | 1 | 0.002 | 1.491 | 1.162 | 1.913 |
|  | ApoB/ApoA1 | WHtR | 0.301 | 0.106 | 8.152 | 1 | 0.004 | 1.352 | 1.099 | 1.662 |
|  | Atherogenic Index | WHtR | 0.169 | 0.080 | 4.416 | 1 | 0.036 | 1.184 | 1.011 | 1.385 |
| Protein Intake (g/day, energy-adjusted) | LDL-C | Group |  |  | 9.541 | 2 | 0.008 |  |  |  |
|  |  | Group (strength) | 1.602 | 0.695 | 4.715 | 1 | 0.030 | 4.961 | 1.270 | 19.368 |
|  | HDL-C | WHtR | -0.182 | 0.084 | 5.009 | 1 | 0.025 | 0.834 | 0.759 | 1.734 |
|  | TC/HDL-C | WHtR | 0.415 | 0.129 | 10.280 | 1 | 0.001 | 1.514 | 1.175 | 1.951 |
|  | LDL-C/HDL-C | WHtR | 0.459 | 0.140 | 10.694 | 1 | 0.001 | 1.582 | 1.202 | 2.083 |
|  | ApoB/ApoA1 | WHtR | 0.326 | 0.111 | 8.657 | 1 | 0.003 | 1.385 | 1.115 | 1.720 |
|  | Atherogenic Index | Energy intake (kcal/day) | 0.000 | 0.000 | 5.675 | 1 | 0.017 | 1.000 | 1.000 | 1.001 |
|  |  | WHtR | 0.194 | 0.087 | 4.961 | 1 | 0.026 | 1.214 | 1.024 | 1.440 |
| Fat Intake (g/kg) | LDL-C | WHtR | 0.216 | 0.084 | 6.576 | 1 | 0.010 | 1.241 | 1.052 | 1.463 |
|  |  | Group (strength) | 1.600 | 0.693 | 5.331 | 1 | 0.021 | 4.955 | 1.274 | 19.281 |
|  | HDL-C | WHtR | -0.166 | 0.078 | 4.527 | 1 | 0.033 | 0.847 | 0.727 | 0.987 |
|  | ApoB | Group |  |  | 12.045 | 2 | 0.002 |  |  |  |
|  |  | Group (strength) | 1.382 | 0.662 | 4.363 | 1 | 0.037 | 3.983 | 1.089 | 14.567 |
|  | TC/HDL-C | WHtR | 0.378 | 0.120 | 9.892 | 1 | 0.002 | 1.460 | 1.153 | 1.848 |
|  | LDL-C/HDL-C | WHtR | 0.398 | 0.126 | 10.011 | 1 | 0.002 | 1.489 | 1.164 | 1.905 |
|  | ApoB/ApoA1 | WHtR | 0.284 | 0.100 | 8.051 | 1 | 0.005 | 1.329 | 1.092 | 1.617 |
|  | Atherogenic Index | Fat intake (g/kg) | 0.710 | 0.273 | 6.749 | 1 | 0.009 | 2.033 | 1.190 | 3.474 |
|  |  | WHtR | 0.190 | 0.081 | 5.465 | 1 | 0.019 | 1.209 | 1.031 | 1.418 |
| Fat Intake (% of energy) | LDL-C | Group |  |  | 10.001 | 2 | 0.007 |  |  |  |
|  |  | Group (strength) | 1.728 | 0.688 | 6.307 | 1 | 0.012 | 5.627 | 1.461 | 21.668 |
|  | HDL-C | WHtR | -0.158 | 0.078 | 4.118 | 1 | 0.042 | 0.854 | 0.733 | 0.995 |
|  | ApoB | Group |  |  | 11.848 | 2 | 0.003 |  |  |  |
|  |  | Group (strength) | 1.486 | 0.658 | 5.096 | 1 | 0.024 | 4.419 | 1.216 | 16.052 |
|  | TC/HDL-C | WHtR | 0.369 | 0.116 | 10.141 | 1 | 0.001 | 1.446 | 1.152 | 1.815 |
|  | LDL-C/HDL-C | WHtR | 0.384 | 0.120 | 10.165 | 1 | 0.001 | 1.468 | 1.159 | 1.859 |
|  | ApoB/ApoA1 | WHtR | 0.267 | 0.100 | 7.125 | 1 | 0.008 | 1.307 | 1.074 | 1.590 |
|  | Atherogenic Index | WHtR | 0.166 | 0.078 | 4.556 | 1 | 0.033 | 1.181 | 1.014 | 1.375 |
| Fat Intake (g/day, energy-adjusted) | LDL-C | Group |  |  | 10.278 | 2 | 0.006 |  |  |  |
|  |  | Group (strength) | 1.609 | 0.698 | 5.317 | 1 | 0.021 | 4.997 | 1.273 | 19.617 |
|  | ApoB | Group |  |  | 12.026 | 2 | 0.002 |  |  |  |
|  |  | Group (strength) | 1.365 | 0.665 | 4.217 | 1 | 0.040 | 3.916 | 1.064 | 14.409 |
|  | TC/HDL-C | WHtR | 0.362 | 0.120 | 9.177 | 1 | 0.002 | 1.437 | 1.137 | 1.817 |
|  | LDL-C/HDL-C | WHtR | 0.380 | 0.125 | 9.209 | 1 | 0.002 | 1.462 | 1.144 | 1.869 |
|  | ApoB/ApoA1 | WHtR | 0.265 | 0.103 | 6.662 | 1 | 0.010 | 1.303 | 1.066 | 1.593 |
|  | Atherogenic Index | Energy | 0.000 | 0.000 | 6.598 | 1 | 0.010 | 1.000 | 1.000 | 1.001 |
| Carbohydrate Intake (g/kg) | LDL-C | Group |  |  | 10.434 | 2 | 0.005 |  |  |  |
|  |  | Group (strength) | 1.673 | 0.689 | 5.887 | 1 | 0.015 | 5.326 | 1.379 | 20.566 |
|  | HDL-C | WHtR | -0.164 | 0.077 | 4.504 | 1 | 0.034 | 0.849 | 0.730 | 0.988 |
|  | ApoB | Group |  |  | 11.643 | 2 | 0.003 |  |  |  |
|  |  | Group (strength) | 1.419 | 0.659 | 4.645 | 1 | 0.031 | 4.135 | 1.137 | 15.033 |
|  | TC/HDL-C | WHtR | 0.371 | 0.117 | 9.974 | 1 | 0.002 | 1.449 | 1.151 | 1.824 |
|  | LDL-C/HDL-C | WHtR | 0.387 | 0.122 | 10.031 | 1 | 0.002 | 1.472 | 1.159 | 1.870 |
|  | ApoB/ApoA1 | WHtR | 0.275 | 0.098 | 7.868 | 1 | 0.005 | 1.317 | 1.086 | 1.596 |
|  | Atherogenic Index | Carbohydrate intake (g/kg) | 0.116 | 0.057 | 4.220 | 1 | 0.040 | 1.123 | 1.005 | 1.255 |
|  |  | WHtR | 0.187 | 0.078 | 5.341 | 1 | 0.021 | 1.198 | 1.028 | 1.397 |
| Carbohydrate Intake (% of energy) | LDL-C | Group |  |  | 9.493 | 2 | 0.009 |  |  |  |
|  |  | Group (strength) | 1.715 | 0.689 | 6.199 | 1 | 0.013 | 5.558 | 1.441 | 21.442 |
|  | HDL-C | WHtR | -0.157 | 0.078 | 4.075 | 1 | 0.044 | 0.854 | 0.733 | 0.995 |
|  | ApoB | Group |  |  | 11.138 | 2 | 0.004 |  |  |  |
|  |  | Group (strength) | 1.461 | 0.657 | 4.941 | 1 | 0.026 | 4.310 | 1.189 | 15.627 |
|  | TC/HDL-C | WHtR | 0.358 | 0.116 | 9.502 | 1 | 0.002 | 1.430 | 1.139 | 1.795 |
|  | LDL-C/HDL-C | WHtR | 0.375 | 0.121 | 9.594 | 1 | 0.002 | 1.455 | 1.148 | 1.846 |
|  | ApoB/ApoA1 | WHtR | 0.280 | 0.103 | 7.449 | 1 | 0.006 | 1.324 | 1.082 | 1.619 |
|  | Atherogenic Index | WHtR | 0.162 | 0.078 | 4.319 | 1 | 0.038 | 1.176 | 1.009 | 1.370 |
| Carbohydrate Intake (g/day, energy-adjusted) | LDL-C | Group |  |  | 9.301 | 2 | 0.010 |  |  |  |
|  |  | Group (strength) | 1.594 | 0.698 | 5.220 | 1 | 0.022 | 4.924 | 1.254 | 19.333 |
|  | ApoB | Group |  |  | 10.929 | 2 | 0.004 |  |  |  |
|  |  | Group (strength) | 1.354 | 0.665 | 4.146 | 1 | 0.042 | 3.872 | 1.052 | 14.254 |
|  | TC/HDL-C | WHtR | 0.365 | 0.121 | 9.143 | 1 | 0.002 | 1.440 | 1.137 | 1.825 |
|  | LDL-C/HDL-C | WHtR | 0.384 | 0.126 | 9.240 | 1 | 0.002 | 1.468 | 1.146 | 1.881 |
|  | ApoB/ApoA1 | WHtR | 0.274 | 0.104 | 6.958 | 1 | 0.008 | 1.315 | 1.073 | 1.612 |
|  | Atherogenic Index | Energy | 0.000 | 0.000 | 6.419 | 1 | 0.011 | 1.000 | 1.000 | 1.001 |

OR, odds ratio; LDL-C, low-density lipoprotein cholesterol; HDL-C, high-density lipoprotein cholesterol; WHtR, waist-to-height ratio; ApoB, Apolipoprotein B; TC, total cholesterol; TG, triglycerides; VLDL-C, very low-density lipoprotein cholesterol; ApoA-1, Apolipoprotein A-1; Lp(a), lipoprotein (a); CHO:PRO, carbohydrate:protein ratio.

Odds ratios (OR) and 95% confidence intervals (CI) are shown.

After Benjamini–Hochberg FDR within models, conclusions were unchanged.
